# GOFFA: Gene Ontology For Functional Analysis – A FDA Gene Ontology Tool for Analysis of Genomic and Proteomic Data

**DOI:** 10.1186/1471-2105-7-S2-S23

**Published:** 2006-09-26

**Authors:** Hongmei Sun, Hong Fang, Tao Chen, Roger Perkins, Weida Tong

**Affiliations:** 1 Z-tech Corporation, 3900 NCTR Road, Jefferson, Arkansas, 72079 USA; 2 National Center for Toxicological Research, Food and Drug Administration, 3900 NCTR Road, Jefferson, Arkansas, 72079 USA

## Abstract

**Background:**

Gene Ontology (GO) characterizes and categorizes the functions of genes and their products according to biological processes, molecular functions and cellular components, facilitating interpretation of data from high-throughput genomics and proteomics technologies. The most effective use of GO information is achieved when its rich and hierarchical complexity is retained and the information is distilled to the biological functions that are most germane to the phenomenon being investigated.

**Results:**

Here we present a FDA GO tool named Gene Ontology for Functional Analysis (GOFFA). GOFFA first ranks GO terms in the order of prevalence for a list of selected genes or proteins, and then it allows the user to interactively select GO terms according to their significance and specific biological complexity within the hierarchical structure. GOFFA provides five interactive functions (Tree view, Terms View, Genes View, GO Path and GO TreePrune) to analyze the GO data. Among the five functions, GO Path and GO TreePrune are unique. The GO Path simultaneously displays the ranks that order GOFFA Tree Paths based on statistical analysis. The GO TreePrune provides a visual display of a reduced GO term set based on a user's statistical cut-offs. Therefore, the GOFFA visual display can provide an intuitive depiction of the most likely relevant biological functions.

**Conclusion:**

With GOFFA, the user can dynamically interact with the GO data to interpret gene expression results in the context of biological plausibility, which can lead to new discoveries or identify new hypotheses.

**Availability:**

GOFFA is available through ArrayTrack software

.

## Background

DNA microarray technology is a key application in pharmaco- and toxicogenomics, a field identified in the U.S. Food and Drug Administration (FDA) Critical Path Initiative  as a major opportunity for advancing medical product development and personalized medicine. It is expected that the review of microarray-based medical devices and microarray data will become an essential regulatory responsibility for the FDA. A single microarray experiment generates a large volume of data and the *management*, *analysis *and *interpretation *of this data challenge both sponsors and regulatory reviewers. Realizing that the integration of these three essential components into one single application will help to realize the full value of this exciting technology, FDA's National Center for Toxicological Research(NCTR/FDA) developed ArrayTrack[1,2], a FDA free bioinformatics resource providing an integrated solution to manage, analyze, and interpret microarray data and the extension to systems biology data. ArrayTrack has been utilized by FDA for the review of genomic data submissions .

The primary emphasis of ArrayTrack is the direct linking of analysis results with functional information for facilitating the interaction between the choice of analysis methods and the biological relevance of analysis results. By selecting one of the analysis methods, the ArrayTrack user can directly link analysis results with functional information such as biological pathways and gene ontology. GOFFA (Gene Ontology For Functional Analysis) is the primary biological interpretation tool using Gene Ontology (GO) [3,4] in ArrayTrack.

GO contains a complex and rich information, posing a challenge in developing statistical and visualization tools to effectively/efficiently utilize and present the information. Many approaches have been investigated to facilitate interpretation of gene expression data using the GO resource [5-18]. Most freely available GO tools are documented on the GO website . These tools are useful to browse and view the GO context when interpreting genomic and proteomic data. However, some do not provide text-annotated GO tree structures (e.g., GoSurfer1.1), or do not retain the fundamental GO hierarchical tree structure (e.g., GoStat, EASE, Onto-Express), or are only microarray specific (e.g., Ontology Traverser), or has operating system dependency limitation (e.g., GOSurfer1.1). Khatri et al [19], Zeeberg et al [3] and Zhang et al [10] did extensive comparisons of various GO-based tools.

Statistical analysis and visualization capabilities are the most important components of any GO tool. Statistical analysis is focused on determining the significant or enriched GO terms. The hypergeometric distribution [20,21], chi-square [22] and Fisher's exact test [5] are three most commonly used enrichment methods. Recently, the Relative Enrichment Factor is also introduced by Zeeberg et al [5]. Reducing GO information to a comprehendible subset based only on statistics alone is unsatisfactory without the aid of visualization. Thus, visualization of the GO hierarchy becomes another important part of the functionality for a useful GO tool. It is highly desirable that a complex query can be directly made to the visually displayed tree to fully integrate statistics and visualization for efficiently mining the GO data.

Here we report the GOFFA software that is designed to further the ability of utilizing GO for interpreting microarray data. GOFFA provides most commonly used statistical functions in an interactive and user-friendly environment. Two effective functions in particular, GO Path and GO TreePrune, were implemented in GOFFA. Unlike other statistical methods that consider each GO term separately by ignoring the hierarchical nature of GO in the enrichment analysis, GO Path identifies the significant terms based on the GO hierarchical tree path using the Fisher's inverse Chi-Squared method [23,24]. GO TreePrune is an interactive tool providing statistical means to adjust and reduce the complexity of GO hierarchical tree information in the form of the node-like visualization. As an integrated component of ArrayTrack, GOFFA has been used in the FDA to interpret both genomic and proteomic data submitted by the sponsors through the Voluntary Genomics Data Submission (VGDS) mechanism .

## Methods

**Figure 1:**
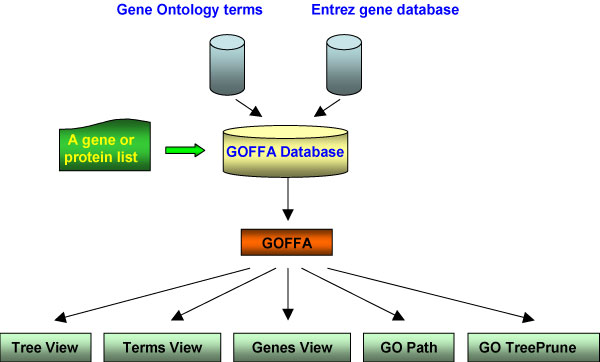
Schematic overview of GOFFA's data flow. GO terms from the Gene Ontology project and gene identifiers from the Entrez Gene databases are combined and linked in the GOFFA database. Lists of genes or proteins from an experiment are analyzed by five functional modules, Tree View, Terms View, Genes View, GO Path and GO TreePrune.

GOFFA's core programming is based on the client-server model. The client is written in JAVA, runs on platforms with the Java run-time environment 1.4 or higher. The server is ORACLE. GOFFA is an integrated component of ArrayTrack, but also can be operated as an independent tool. Figure 1 shows the program logical structure.

### GOFFA Database

GOFFA uses an ORACLE database containing the GO project data together with gene identifiers for human, mouse and rat from the NCBI Entrez gene database . The database currently contains 16,389 mouse, 11,934 human and 11,599 rat genes. The genes from these three species can also be combined in analyses using the "cross-products" feature, where the same gene symbols from human, mouse and rat (regardless of case) are considered to share the same functional annotation in GO; in this case, the GOFFA database contains 26,564 unique gene symbols for cross species annotation.

### GOFFA Tree

Data from the GO website are downloaded in a structure called a directed acyclic graph (DAG), a name that denotes an unclosed structure where a particular child node associated with a GO term can have multiple parent nodes. GOFFA converts the DAG structure to a tree structure by constructing distinct paths from the highest parent node (least specific), successively down through progeny to the lowest (most specific) child node. In converting the data, GOFFA maintains the GO database's so-called true path rule by assuring that a gene product GO term applicable to a child node also applies to all parent terms. Thus, during the conversion to a tree structure, the DAG structure for each GO term can become many separate traversals from highest parent to lowest child. Each such traversal in GOFFA is called a GOFFA Tree Path, and each node along a GOFFA Tree Path is assigned a unique identification called a GOFFA ID. Consequently, the same GO term occurring in different GOFFA Tree Paths has a distinct GOFFA ID in each path. The restructuring of GO information in the GOFFA Tree Path format not only markedly speed up database queries but, most importantly, enable developing two unique utilities, GO Path and GO TreePrune (more in Results).

### Statistical Analysis

A fundamental step in analyzing DNA microarray data is to determine the differentially expressed genes (DEGs) for subsequent biological interpretation. GOFFA applies three statistical approaches to determine the significant GO terms for a given list of DEGs, two previously reported methods and one novel approach:

• Fisher's Exact Test – A right-sided Fisher's Exact Test  is used to estimate the statistical significance of GO term *i*. Four lists of genes are needed to calculate the significance (i.e., p-value): The number of inputted genes (*M*) [25], the subset of *M *genes that belong to GO term *i *(*m*_*i*_), the set of reference genes (*N*) and the subset of *N *genes that belong to GO term *i (_*ni*_)*. The accuracy of p-value is largely dependent on the choice of the set of reference genes. There are two options in GOFFA to determine *N*, depending on whether the genes derived from a known gene or not. For a known microarray chip, *N *is the total number of genes on the chip; in this case, p-value less than 0.01 normally is indicative of a statistically significant finding. GOFFA provides information associated with most commercial array platforms, including most GeneChip platforms from Affymetrix, most one- and two-channel array platforms from Agilent, as well as numerous other array platforms such as those from GE HealthCare CodeLink, Illumina BeadArray, and Applied Biosystems arrays, etc. If the microarray chip's genes are unknown, the total number of genes in the GOFFA database is assigned as *N*. In this case, the choice of *N *is dependent on the selected species, currently, 16,389 genes for mouse, 11,934 for human, 11,599 for rat, and 26,564 if combining all three species. Thus, the selection of *N *is an important factor to interpret p-value.

• Relative Enrichment Factor – GOFFA also calculates the Relative Enrichment Factor (E) for assessing the significance of GO term *i *for a given list of DEGs [5]. The E-value is calculated as:

E = (*m*_*i*_/*M*)/(*n*_*i*_/*N*)     (1)

where *m*_*i*_, *M*,* n*_*i *_and *N *are defined the same as for Fisher's Exact Test described in the preceding paragraph. E provides a direct measure of the prevalence of a GO term *i *among the *M *significant genes compared to the prevalence of the same GO term *i *among *N *total genes. Accordingly, E = 1.0 corresponds to GO term *i *occurring among the DEGs at the same prevalence as among the *N *total genes. E = 2.0 indicates that GO term *i *occurring in the DEGs two times more than occurring in the *N *total genes, indicating significant findings.

• GOFFA Tree Path Ranking – Criteria based on Fisher's Exact Test and/or Relative Enrichment Factor sometimes fail to sufficiently condense and clarify results for effective interpretation, especially for large lists of significant genes. This provided the motivation of developing this unique function in GOFFA. The method applied the Fisher's inverse Chi-Squared method [23,24] to sort GOFFA Tree Paths in accordance with their likely significance, and then renders an interactive graphic display for visualization and interpretation. The Fisher's inverse Chi-Square method uses the fact that given a uniform distributed p-value, -2log(*p*) has a chi-square distribution with two degrees of freedom, and hence the statistic



follows a chi-square distribution with 2*K *degrees of freedom when the joint null is true. In our case, *p*_*k *_is the Fisher's Exact Test probability value of GO term *k *and *K *is a total number of GO terms within the traverse of the GOFFA Tree Path from the upper level of the tree down to GO term *i*. Thus, *R*_*i *_is a relative metric of the prevalence of a GOFFA Tree Path from the upper level to the level GO term *i *belongs, given that the *p*_*k *_values are known for each GO term on the path. The greater the value of *R*_*i*_, the less likely it is that the significance of a GOFFA Tree Path is a chance occurrence.

### Availability

GOFFA is available through ArrayTrack software .

## Results

### GOFFA Features

**Figure 2:**
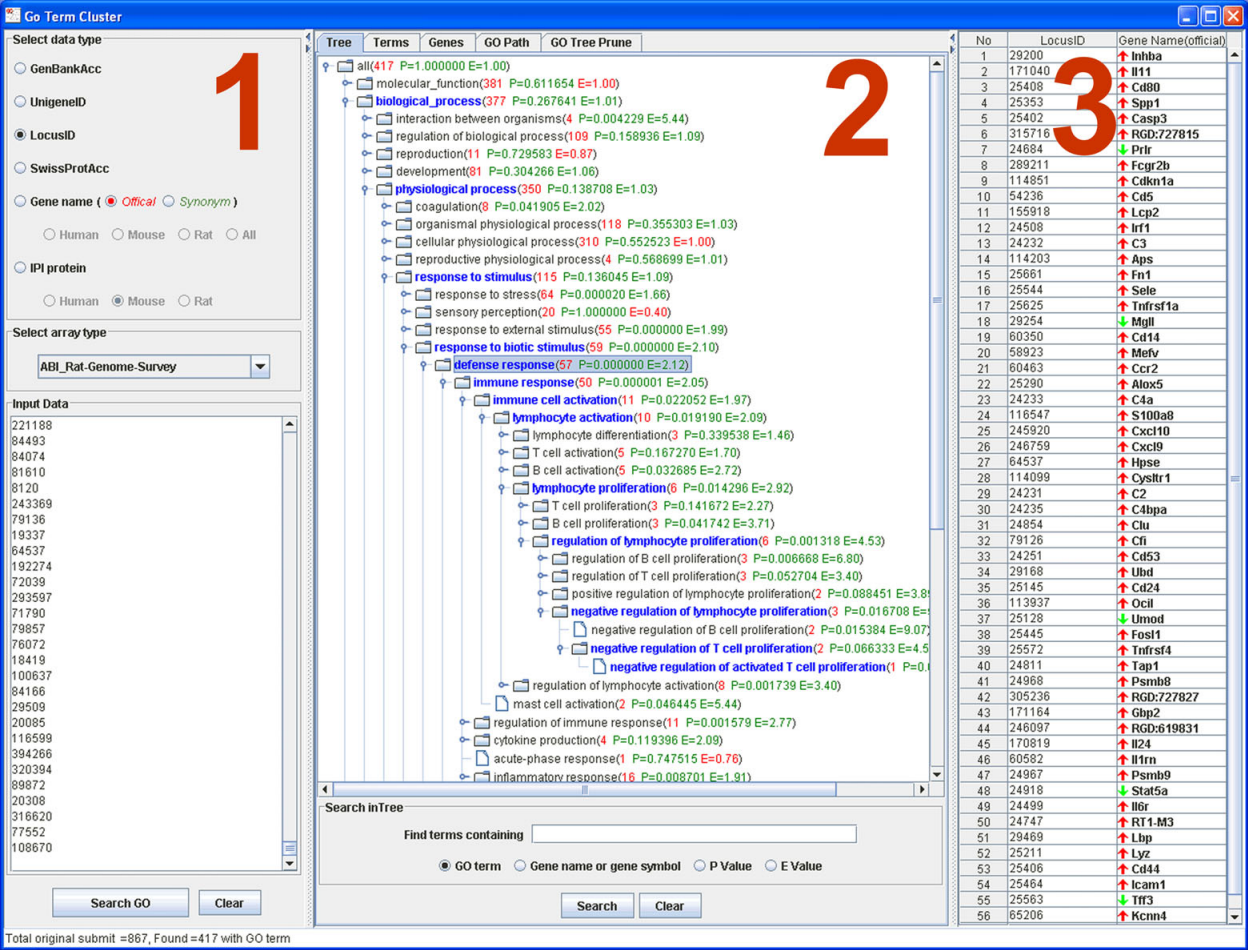
GOFFA interface and Tree Window – The GOFFA interface contains three panels: the left panel (labeled 1) is for queries, the center panel (labeled 2) for tabular and/or graphical displays of and for interaction with the GO information, and the right panel (labeled 3) lists the individual genes associated with the GO information presented in the center panel. The displayed Tree Window in the center panel is the default view of GOFFA, which enables the hierarchical display of the GO terms in a outline-like tree format; p- and E-values as well as the number of genes are also displayed for each GO term. E-values >1 are shown in green and those <1 in red, respectively denoting greater or lesser prevalence, respectively, of the GO term in the inputted gene list rather than in the overall experimental platform. The user can query the tree by GO term, gene name/symbol, p-value, E-value and in combination with functions below the view. The query-match GO terms are highlighted as blue.

The GOFFA's software GUI, shown in Figure 2, has three panels with different functions that are designed for intuitive and interactive use. The left panel (labeled 1) is for queries, the center panel (labeled 2) for tabular and/or graphical displays of and for interaction with the GO information, and the right panel (labeled 3) lists the individual genes associated with the GO information presented in the center panel.

Queries are initiated in the left panel by pasting DEG ID's into the query window, one gene per line. The input gene ID's must correspond to the "Select data type" option chosen by the user. Currently, GOFFA supports four types of gene identifiers: (1) GenBank Accession number, (2) Unigene ID, (3) LocusLink ID (or Entrez Gene ID) and (4) Gene Name. In addition, GOFFA supports two protein identifiers, IPI ID (EBI International Protein Index database) and Swiss-Prot accession number for proteomics data analysis. The GOFFA database currently contains GO annotation data for 105 microarray platforms that, with the "Select array type" option, is coupled with the GO analysis and available for display. Query results are displayed in the center panel in five interactive viewing windows, Tree, Terms, Genes, GO Path and GO TreePrune, that are activated with tabs at the top of the panel. These five windows provide the means for applying and iteratively re-applying statistical operators to the inputted (DEG) gene list, viewing statistical results, and viewing the results of GOFFA's Tree, GO Path, and GO PruneTree analysis. The data and results within both tables and plots are synchronized components, enabling mouse-click toggling between window views. For example, genes associated with GO terms that are selected through mouse clicks in the GOFFA Tree (panel 2, Figure 2) are displayed as a list in the right panel (panel 3, Figure 2).

**Figure 3:**
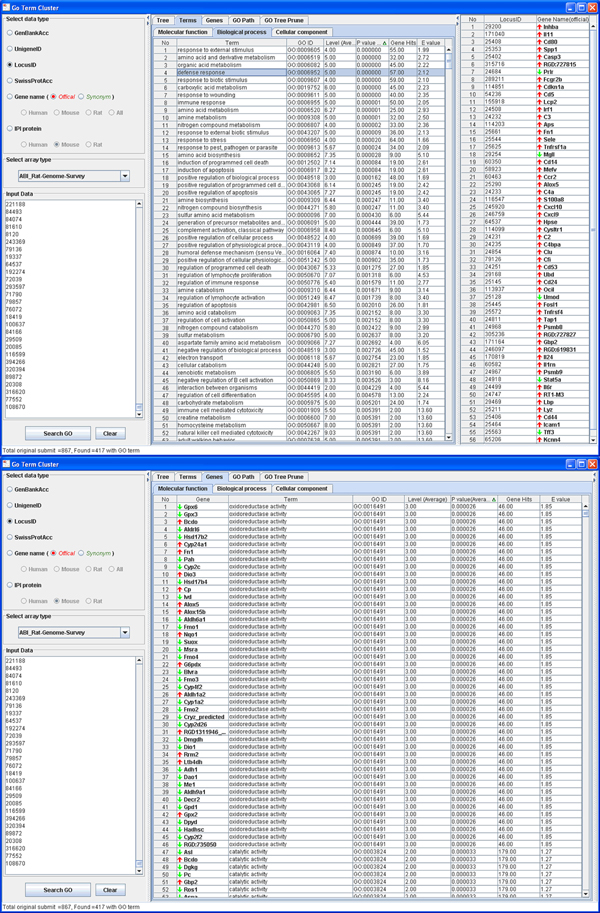
Terms and Genes Windows – The Terms Window (A) and Genes Window (B) summarize the findings associated with GO terms and genes respectively in the tabular format along with various statistical parameters (e.g., p- and E-values). Each View contains three tables corresponding to three categories of GO (molecular functions, biological processes and cellular components). The table can be sorted in every column by clicking on the column header. Sorting on multiple columns is also supported (pressing Ctrl key while clicking on the second column header for sorting). Both copy/paste and export functions are available to transfer data to external software.

**Figure 4:**
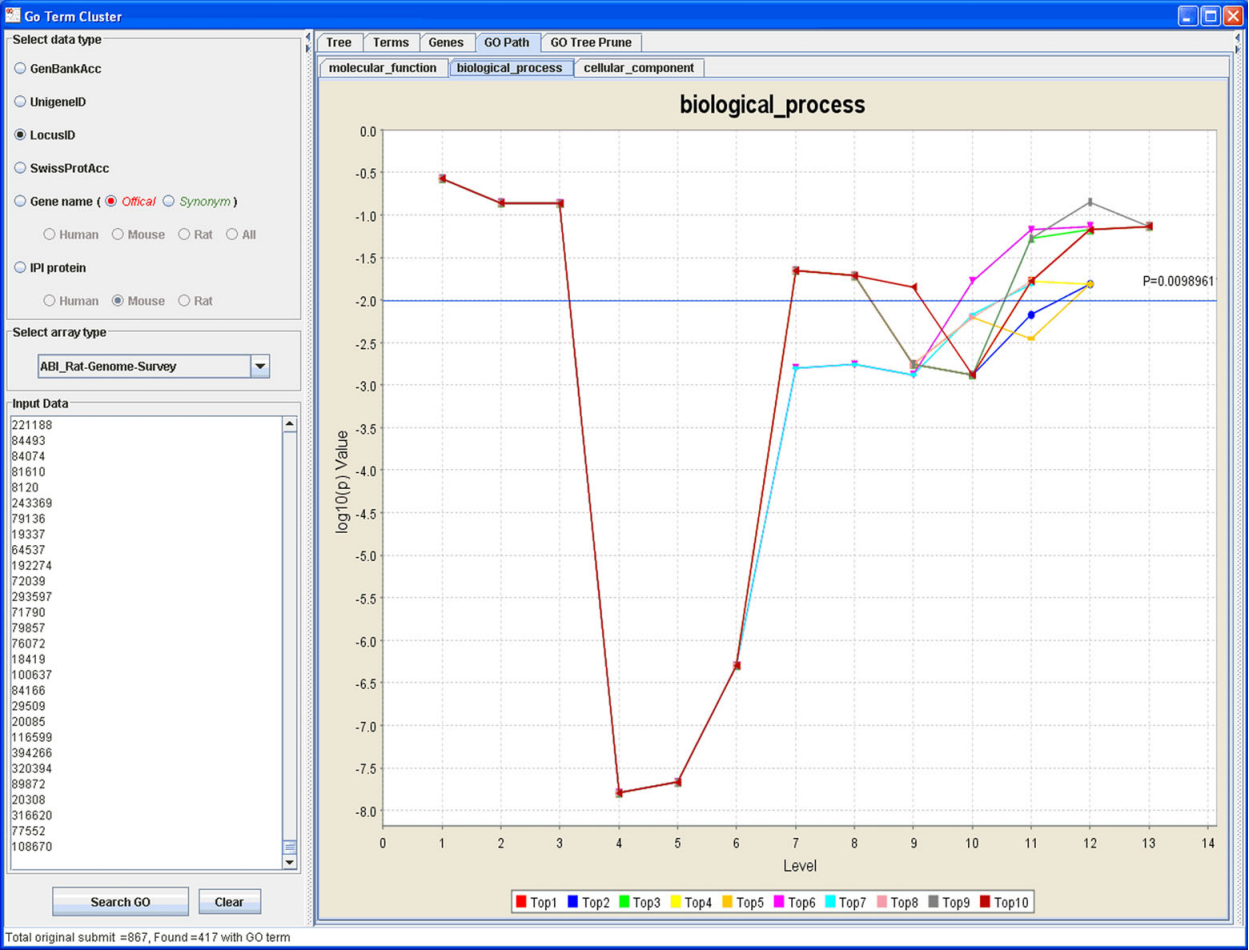
GO Path – GO Path sorts, by descending statistical significance based on an inverse Chi-Squared test, the GOFFA Tree Paths (i.e., linked GO terms) and graphically displays them from high to low at each hierarchical level. GO Path plots the top ten paths with solid circles representing the GO terms on the path. The X-axis has the hierarchical level to which the GO term belongs and the Y-axis (log p) indicates the statistical significance of the term. A color key for the top 10 paths (as determined by equation 2) is located beneath the plot. Clicking either a circle in a path in the plot or its corresponding color key launches a Tree View (Figure 2) with the selected path highlighted in blue. Other features are also available from a popup menu obtained by right clicking the plot, including zoom in/out, export figure, etc.

**Figure 5:**
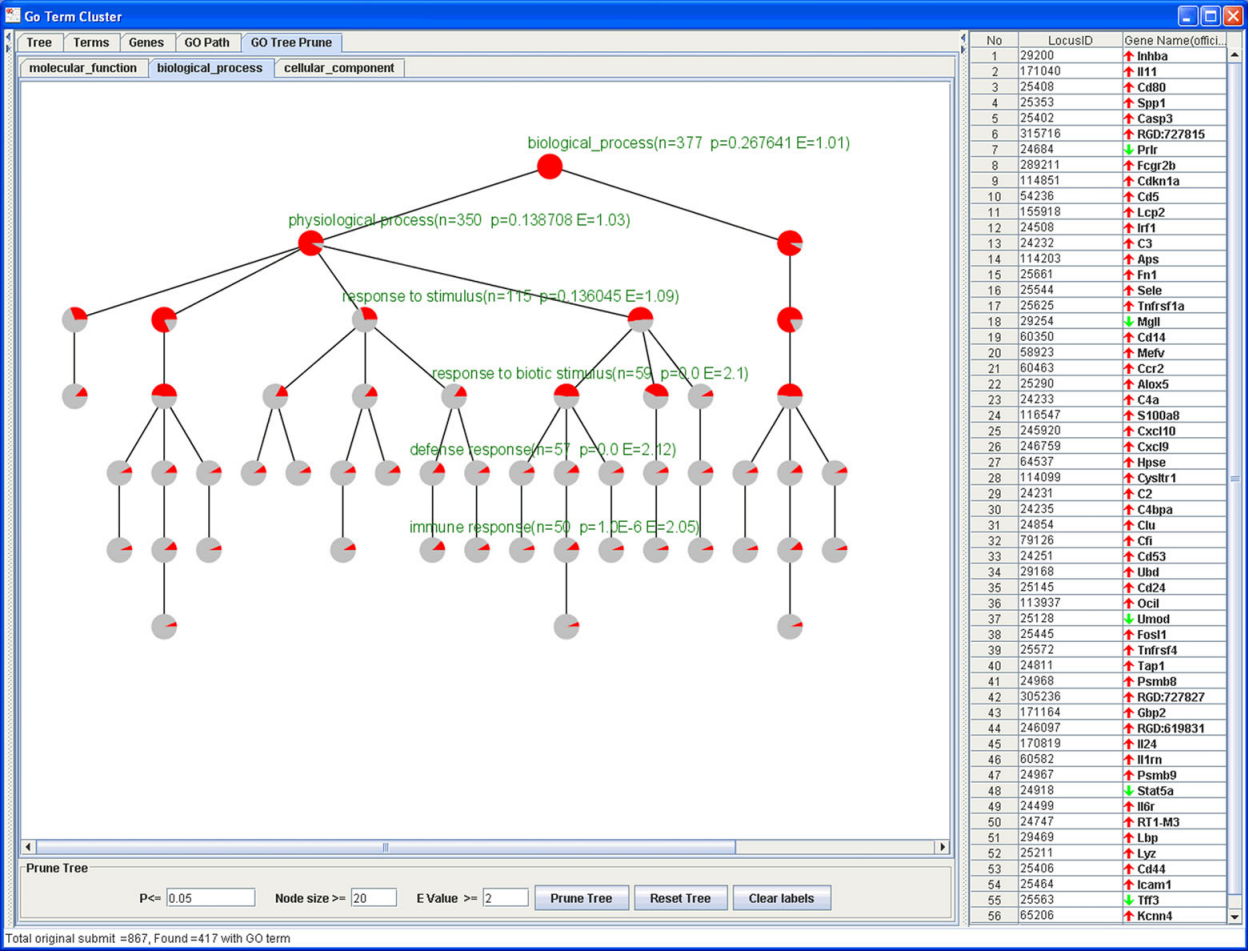
GO TreePrune – This node-like tree display allows the user to filter out nodes and thus reduce the complexity of a tree by specifying the p- and E-value as well as the user-defined number of genes in the end node. A GO term is represented by a sectored pie, where the red sector shows the percentage of the inputted genes associated with the term. The individual genes associated with each term are displayed in the right panel by single clicking the term. The annotation of a term can be turned on or off by double clicking the term. Each term is movable with mouse drag, which is convenient when working on a dense tree or with many annotations. The tree diagram can be zoomed and moved by holding down the right or left button of the mouse, respectively.

The user can toggle between the center panel's five windows (panel 2, Figure 2), providing another level of iterative interactivity. Each window either displays information differently, or displays different information related to the inputted genes:

• Tree window – The Tree window is the default viewer that is launched after a search, and appears in the center panel. As shown in Figure 2, the Tree window displays GO terms in an outline-like hierarchical tree format (conventional view). The number of associated genes, the Relative Enrichment Factor (E-value), and the p-value from the Fisher's Exact Test are displayed for each GO Term at each GO hierarchical level. Since query results can form an extensive list, a flexible search capability is provided below the tree display. The user can search the tree by GO term, gene name/symbol, p-value, E-value, and their combination, and search results are then highlighted in blue within the display for easy location with associated gene (s) listed in the right panel.

• Terms and Genes windows – These two windows provide alternative, tabular presentations of the information contained in the tree window (Figure 3). Whereas the Tree window combines the three categories of GO path information, both the Terms window (Figure 3a) and Genes window (Figure 3b) separately display Molecular Function, Biological Process and Cellular Component category information, as chosen with a tab, and presents it in an excel-like spreadsheet format. As indicated by their names, the Terms window aggregates information by individual GO term, whereas the Genes window aggregates information by individual gene. Both windows display results of statistical operators (p-value and E-value). The Terms window displays the number of significant genes associated with each GO term, as well as the average hierarchical level at which the gene appears in the GO term. Tables in both windows can be sorted in either ascending or descending order of any column, and can be cut and pasted or exported to external software for further analysis.

• GO Path window – The GOFFA GO Path plot (Figure 4) visually presents the GOFFA Tree Paths estimated as the most relevant by equation 2. The GOFFA algorithm first rank-orders all GOFFA Tree Paths using equation 2 values, and then plots the 10 paths with the highest values, with the X-axis corresponding to descending hierarchical tree level, and the Y-axis corresponding to the log p value at each hierarchical level (Figure 4). Double clicking any GOFFA Tree Path in the graph or its color key located below the graph will launch a Tree window view (Figure 2, panel 2) with the GO terms corresponding to the GOFFA Tree Path highlighted in blue for easy recognition. The GO Path visualization could be considered as a condensed rendering of the most salient GO information associated with the DEG's data.

• GO TreePrune – This visualization tool display the GO terms in a node-like hierarchical tree structure, as shown in the Figure 5 example. Note that the plot is annotated with the p-value, E-value, and number of associated genes at each node of the tree. The number of genes associated with each node is also depicted in the pie chart as a fraction of the genes associated with the root node. The GO TreePrune plots can be very large and complex; as a result, GOFFA provides a tool for pruning the tree by assigning arbitrary and simultaneous cutoffs for p-value, E-value, and number of genes. Nodes below the cutoff values specified by the user are removed from the plot.

### GOFFA Application

A dataset from a toxicogenomics study was used to demonstrate the utility of GOFFA. In this study, the renal toxicity and carcinogenicity associated with the treatment of aristolochic acid (AA) in rats was studied using DNA microarray. AA is an active component of herbal drugs derived from some plants that has been used for medicinal purposes since ancient time [26]. The compound is a nephrotoxin and carcinogen in human and rodents. To investigate the effect of AA exposure on gene expression in rat kidney, a toxicogenomics study is conducted; the experimental protocol is described by Chen et al. in an accompanying paper of the same issue. Briefly, six-week old Big Blue rats were treated with AA and control vehicle for 3 month. One day after the last treatment, the animals were sacrificed and the kidneys were removed for microarray analysis using the Applied Biosystems Rat Genome Survey Microarray. Both treated and control samples had six biological replicates (rats). The data normalization and analysis were conducted using ArrayTrack. The DEG list was determined based on p < 0.01 and Fold Change > 2. Since GOFFA is fully integrated with ArrayTrack, the DEGs from ArrayTrack were directly passed to GOFFA for functional analysis. Of 1176 identified genes, 417 genes had GO information for analysis [25]. The GOFFA results are summarized in Figures 2, 3, 4, 5.

The statistics based on a combination of Fisher's Exact Test (p < 0.05) and Relevant Enrichment Factor (E > 2) identified 52 enriched GO terms in the GO biological process. The majority of the terms are related to four functional categories, induction of apoptosis, defense response, response to stress, and amino acid metabolism. These four functional categories reflect the known biological and pharmacological responses of kidney to the AA treatment [26]. Out of these four functional categories, GO Path ranked "defense response" as an important mechanism associated with the AA treatment (Figure 4), and similar results were obtained from GO TreePrune as well (Figure 5). This finding is consistent with the general understanding that defense response, which includes immune response, is a complex network response of a tissue to toxins and carcinogens (such as AA) for defending the body. Figure 2 gives the GO Path results in the Tree window, where the majority of genes involved in the defense response are up-regulated to oppose damage by AA. For example, the *inhba *gene (first gene in the right panel) is a growth factor with 4.1-fold increase in expression in kidney. This is a tumor-suppressor gene and it produces protein that increases arrest in the G1 phase of tumor cells [27]. Therefore, its induction inhibits tumorigenesis in kidney treated with AA.

## Discussion

A fundamental step in analyzing DNA microarray data is to determine the differentially expressed genes (DEGs) that are presumably relevant to the biological phenomena under study. However, in microarray experiments using chips with thousands of genes where a small subset of DEGs is determined for a disease or toxicity, the potential for both type 1 and type 2 errors could be large. Both types of errors suggest the need for the biologists to intervene in the data reduction and analysis process beyond the application of statistics. The GOFFA software was designed with the biologist in mind. The platform provides a means to analyze and scrutinize the complex data from genomics and proteomics experiments in the context of the existing knowledge of gene function as embodied by the GO database. It provides the biologist alternate ways to summarize data, statistically select the most relevant data, or examine in fine detail the biological phenomena associated with selected data.

GOFFA is a client-server application, written in JAVA language for portability, and has a GUI designed with the assistance of biologists for their own intuitive ease of use. The GUI is logically divided into three panels (Figure 2), for queries (panel 1), analysis and results (panel 2), and gene lists (panel 3), respectively. The GO analysis, results tables, graphs, and visualization tools are accessed from the analysis and results panel (Figure 2, panel 2) that maintains data linkage assuring ease in examining selected data in different ways.

GOFFA's efficiency and effectiveness for data interpretation results from treating GO data as a set of distinct hierarchical GOFFA Tree Paths. Application of statistical tests to the GOFFA Tree Paths enables two unique interpretive functions, GO Path and GO TreePrune. GO Path provides the rank ordered estimates of the statistically important GOFFA Tree Paths. GO TreePrune provides the ability to prune GO trees by removing the GO terms according to their p- and E-values in conjunction of the user-defined number of genes the terms contain. These two functions apply the different statistical approaches to rank and/or narrow down the GO terms for further analysis/interpretation. When used together, the functions enable the biologist to reduce complexity of data to that which is most relevant, select that information, and then drill down to examine it further at a more refined level of detail.

The statistical estimators used in GOFFA (as well as other similar GO tools) should be interpreted as heuristic metrics of the potential biological significance of GO terms, rather than formal inferences of biological relevance. They are most reliable for problem solving when all genes from an experiment are known, since the prevalent GO terms in DEG's are compared to the prevalent GO terms in the set of reference genes. For example, the absolute p-value from the Fisher's Exact Test has little value unless the total number of genes on the chip is used as the set of reference genes. This is equally applied to the E-value. GOFFA currently provides gene lists for over 100 commercial array types (e.g., most GeneChip and Agilent's arrays), for which the GO terms are pre-mapped and stored in the database for quick retrieval and analysis. With this information, GOFFA's statistical estimators can provide more meaningful significance assessment for interpretation of the GO results. If the inputted gene list is not associated with an array type, the total numbers of genes in the GOFFA database is for statistical estimates; while this will, for example, unrealistically skew p-values, p-values across the GO terms will still retain meaning in a relative sense.

While GOFFA itself is a powerful analysis tool, its full utility derives from its integration as a module of the ArrayTrack software. ArrayTrack is a comprehensive software platform for microarray data management, analysis and interpretation [1,2]. The integration of GOFFA with ArrayTrack enables the microarray data to be easily processed in the ArrayTrack environment and the resultant DEG list immediately interpret with GOFFA. Importantly, ArrayTrack has been interfaced with various commercial pathway software, providing an additional means to investigate the validity of GOFFA findings with respect to relevant gene ontologies.

## Conclusion

A common characteristic of high-throughput omics technologies, such as DNA microarray, is the generation of huge datasets that provide the ability to examine differential expression between corresponding genes in treatment and control groups. GOFFA enhances the capability to interpret data generated from these technologies. GOFFA applies statistical analysis in conjunction with intuitive visual display to present GO terms, trees and paths in a manner to facilitate biological interpretation. There are two unique tools available in GOFFA, GO Path and GO TreePrune, both enabling fast and interactive interrogation of significant gene and protein lists through statistical assessment and visual inspection. GOFFA is a module of ArrayTrack that is FDA's microarray data management, analysis and interpretation software.

## Authors' contributions

HS has developed GOFFA and finished the first draft of the manuscript. WT conceived the concept of the GO Path function and finalized the manuscript. HF was involved in the GOFFA interface design and testing and contributed significantly on finishing the first draft of the manuscript. TC helped preparing the section for the real-world application of GOFFA. All authors were involved with the design of the GOFFA functions and user interface. All authors participated in preparation of the manuscript, and approved its final form.
